# Sleep disturbances in Japanese patients with inflammatory bowel disease and their impact on disease flare

**DOI:** 10.1186/s40064-016-3408-6

**Published:** 2016-10-13

**Authors:** Risa Uemura, Yasuhiro Fujiwara, Narika Iwakura, Masatsugu Shiba, Kenji Watanabe, Noriko Kamata, Hirokazu Yamagami, Tetsuya Tanigawa, Toshio Watanabe, Kazunari Tominaga, Tetsuo Arakawa

**Affiliations:** 1 Department of Gastroenterology, Osaka City University Graduate School of Medicine, 1-4-3 Asahimachi, Abeno-ku, Osaka, 545-8585 Japan; 2 Department of Gastroenterology, Osaka City General Hospital, 2-13-22 Miyakojima-hondori, Miyakojima-ku, Osaka, 534-0021 Japan

**Keywords:** Crohn’s disease, Disease flare, Inflammatory bowel diseases, Sleep disturbances, Ulcerative colitis

## Abstract

**Background:**

Several studies have reported a significant association between sleep disturbance and inflammatory bowel disease (IBD). The aim of the present study is to compare the clinical characteristics and the health-related quality of life (HR-QOL) of Japanese IBD patients with or without sleep disturbances, and to investigate the risk factors for disease flare in these patients.

**Methods:**

IBD patients were asked to complete a self-administered questionnaire including the Pittsburg sleep quality index and the 8-item short-form health survey. The information about disease flare within 1 year from enrollment in the study was analyzed with a multiple logistic regression model to identify risk factors for IBD flare-ups.

**Results:**

The prevalence of sleep disturbances was 44.1 % (60 out of 136 IBD patients). Use of sleep medications was significantly higher in IBD patients with sleep disturbances whereas use of immuno modulators was significantly higher in IBD patients without sleep disturbances. The scores from all HR-QOL domains were significantly lower in patients with sleep disturbances than in patients without sleep disturbances. Fifty-one patients (37.5 %) had disease flare within 1 year from enrollment in the study and sleep disturbances were identified as a significant risk factor for disease flare (OR 3.09, 95 % CI 1.47–6.43).

**Conclusions:**

Our results indicate that sleep disturbances are common in Japanese IBD patients and are associated with poorer HR-QOL. Since the presence of sleep disturbances is a significant risk factor for IBD flare-ups, we encourage physicians to perform a careful examination of sleep disturbances in IBD patients.

## Background

Inflammatory bowel diseases (IBD) such as Crohn’s disease (CD) and ulcerative colitis (UC) are chronic inflammatory disorders with unknown pathogenesis. Although the prevalence of IBD in Asian countries is lower than that in Western countries (Thia et al. 2008), it has been rapidly increasing in Japan; prevalence of CD in 2014 was 33.4 per 100,000 people and that of UC was 142.9 per 100,000 people. IBD displays waxing and waning phases with periods of asymptomatic remission interrupted by episodes of disease flare. Therefore, the primary treatment goal of IBD is to improve patients’ quality of life (QOL) by treating flare-ups and maintaining remission (Swanson et al. 2011). However, disease flare is thought to be multifactorial (Bernstein et al. 2010), and both triggers and risk factors for disease flare remain unclear.

Sleep disturbances are common clinical conditions and approximately 20 % of Japanese adults experience sleep problems (Liu et al. 2000). Several studies have revealed that sleep disturbances are associated with multiple conditions and diseases. In the gastrointestinal tract, significant associations between sleep disturbances and gastroesophageal reflux disease (Fujiwara et al. 2012), functional dyspepsia (Lacy et al. 2011), irritable bowel syndrome (Ranjbaran et al. 2007a; Nojkov et al. 2010), and IBD (Ranjbaran et al. 2007a; Graff et al. 2011; Ali et al. 2013; Gingold-Belfer et al. 2014; Ananthakrishnan et al. 2013, 2014) have been reported. Sleep disturbances are commonly found in IBD patients, especially those with active disease (Graff et al. 2011; Ali et al. 2013; Gingold-Belfer et al. 2014; Ananthakrishnan et al. 2014), and associated with new onset of UC (Ananthakrishnan et al. 2014) and CD flares (Ananthakrishnan et al. 2013). However, the prevalence of sleep disturbances in Japanese IBD patients and the association between sleep disturbances and disease flare is unknown.

The aim of the present study was to compare the clinical characteristics and health-related QOL (HR-QOL) of IBD patients according to the presence or absence of sleep disturbances, and to investigate the risk factors for disease flare within 1 year from enrollment in the study.

## Methods

### Subjects

Patients were enrolled between June 2012 and August 2013 at the Osaka City University hospital. Adult IBD outpatients were eligible only if they met the inclusion criteria for this study. Only patients who were ≥18 years of age and treated with medicine at our hospital were included in the present study. The exclusion criteria included: (1) shift workers; (2) pregnancy and nursing; (3) severe malignant disease; (4) severe sleep disorders such as obstructive sleep apnea syndrome that required nasal continuous positive airway pressure; and (5) home parenteral nutrition or infusion therapy. Patients with a stoma were also excluded because standard symptom-based measures of disease activity are not applicable to these patients. This study was approved by the Osaka City University Ethics Committee and informed consent was obtained from all participants.

### Questionnaire and data collection

All participants were asked to complete a self-administered questionnaire aimed at evaluating alcohol drinking habits, smoking status, caffeine intake, sleep disturbances, and HR-QOL. Sleep disturbances were assessed by a Japanese version of the Pittsburg sleep quality index (PSQI) while HR-QOL was evaluated according to a Japanese version of the 8-item short-form health survey (SF-8). The demographics and information including age, sex, height, body weight, types of IBD, disease duration, disease activity, presence or absence of prior surgery for IBD, symptoms, current medications, and comorbidities were collected from medical charts. Body mass index (BMI) was calculated as the ratio between body weight and the squared height (kg/m^2^).

### PSQI and definition of sleep disturbances

The PSQI consisted of 17 individual items that generated the seven component scores: subjective sleep quality, sleep latency, sleep duration, habitual sleep efficiency, sleep disturbances, use of sleep medication, and daytime dysfunction. Each component score ranged from 0 to 3, and the sum of all the component scores provided the total score, with high scores indicating poor sleep (Buysse et al. 1989). In the Japanese version, a PSQI >5.5 has a sensitivity of 80.0–85.7 % for several different patient groups and a specificity of 86.6 % for control subjects (Doi et al. 2000). Patients with a total PSQI >5.5 were classified as patients with sleep disturbances (Doi et al. 2000).

### HR-QOL assessment

The SF-8 was developed to estimate HR-QOL based on the scores from eight domains and two summaries: physical functioning (PF), role physical (RF), bodily pain (BP), general health perception (GH), vitality (VT), social functioning (SF), role emotional (RE), mental health (MH), physical component summary (PCS), and mental component summary (MCS) (McHorney et al. 1993; Ware et al. 1994). PF, RF, and BP scales show the strongest correlation with the physical component. The mental component correlates show the strongest correlation with the RE and MH scales. Three of the scales (GH, VT, and SF) have significant correlations with both components. Importantly, SF-8 scores show a strong correlation with SF-36 scores. Scores for PCS and MCS are calculated according to the manual of the Japanese version of the SF-8. A score of ~50 is the average for the Japanese adult population in eight domains and two summaries, while a lower score indicates a poorer HR-QOL (Fukuhara and Suzukamo 2004).

### Disease activity

We evaluated the disease activity using the Harvey-Bradshaw index (HBI) for CD (Harvey and Bradshaw 1980; Vermeire et al. 2010) and the partial Mayo score (pMayo) for UC (Schroeder et al. 1987; Leong et al. 2014; Lewis et al. 2008). HBI was composed of 5 parameters including general well-being (ranged from 0 to 4), abdominal pain (ranged from 0 to 3), number of liquid stools per day (1 per occurrence), abdominal mass (ranged from 0 to 3), and complications (1 per item such as arthralgia, uveitis, erythema nodosum, aphthous ulcer, pyoderma gangrenosum, anal fissure, new fistula, and abscess). The higher scores indicated more severe disease activity and an HBI greater than 5 was used to define an active disease for cases of CD (Vermeire et al. 2010). The pMayo entailed information on diarrhea, rectal bleeding, and a physician’s global assessment that ranged from 0 to 3. Higher pMayo scores represented greater UC disease activity and pMayo scores greater than 3 were used to defined an active disease for UC (Minami et al. 2015).

### Assessment of disease flare

A retrospective cohort study was performed to examine the association between disease flares and sleep disturbances. We investigated the risk factors for disease flares in the year preceding enrolment. The information about disease flare within 1 year from enrollment was assessed by using medical charts. The disease flare was defined as a case with either: (1) more than 2-point increase in disease activity score, (2) cases requiring dose escalation of immunosuppressive therapy such as tacrolimus hydrate, azathioprine, and mercaptopurine, (3) initiation or addition of new drugs such as anti-TNFα biologic agents, (4) treatment with IBD-related surgery, or (5) hospitalization.

### Statistical analysis

Values are presented as mean and standard deviation (SD), frequency (%), or odds ratio (OR) with 95 % confidence intervals (CI). Comparisons between the two groups were performed using the Chi-squared test or the student’s *t* test. A multiple logistic regression model was created to assess independent associations between risk factors and disease flare within 1 year from enrollment. Data were considered statistically significant at P < 0.05. The statistical analysis was performed with SPSS (21.0 J SPSS Japan, Tokyo, Japan).

## Results

### Prevalence of sleep disturbances among patients with IBD

**Fig. 1:**
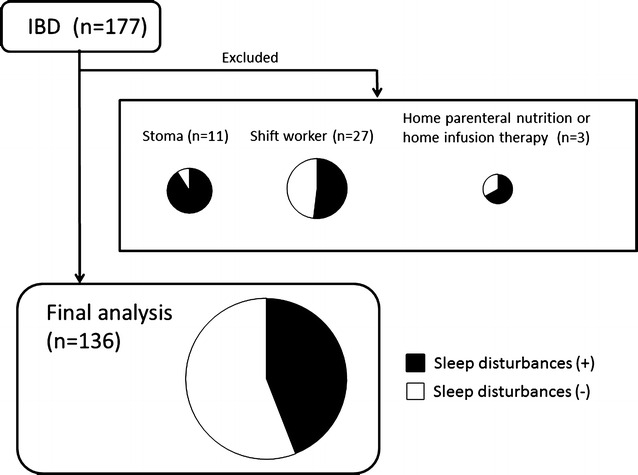
Subjects enrolled in this study and prevalence of sleep disturbances. Among the 177 IBD patients enrolled, 41 patients were excluded: 27 were shift workers, 11 had a stoma, and 3 were undergoing home parenteral nutrition or home infusion therapy. The remaining 136 patients were analyzed in the present study. Prevalence of sleep disturbances in IBD patients was 44.1 % (60 out of 136 patients)

**Fig. 2:**
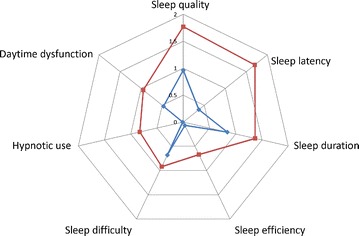
Sleep components scores among IBD patients with and without sleep disturbances. All component scores of the PSQI in IBD patients with sleep disturbances were significantly higher than the scores measured in IBD patients without sleep disturbances. The *red line* and the *blue line* represent the mean component score of patients with sleep disturbances and without sleep disturbances, respectively

A total of 177 IBD patients were enrolled in the present study. We excluded a total of 41 patients: 27 patients were shift workers, 11 had a stoma, and 3 were undergoing home parenteral nutrition or home infusion therapy. The final study was performed on 136 patients (Fig. 1). Sleep disturbances were found in 60 (44.1 %) out of 136 IBD patients. All PSQI component scores such as sleep quality, sleep latency, sleep duration, sleep efficiency, sleep difficulty, hypnotic use, and daytime dysfunction were significantly lower in patients with sleep disturbances compared to those without sleep disturbances (Fig. 2). Among patients who were excluded from the study, sleep disturbances were reported in 10 (90.9 %) out of 11 patients with a stoma, 14 (51.9 %) out of 27 shift workers, and 2 (66.7 %) out of 3 with home parenteral nutrition or infusion therapy (Fig. 1).

### Clinical characteristics of IBD patients with and without sleep disturbances

Clinical characteristics of IBD patients with and without sleep disturbances are shown in Table 1. Use of sleep medications was significantly higher in IBD patients with sleep disturbances whereas use of immuno modulators was significantly higher in IBD patients without sleep disturbances. No significant differences in HBI and pMayo scores between IBD patients with and without sleep disturbances were observed, suggesting that disease activity was not associated with sleep disturbances. There were no significant differences in other factors including age, sex, BMI, alcohol or caffeine intake, smoking, IBD type, presence of active disease, disease duration, prior IBD surgery, and use of steroids, 5-aminosalicylates, and anti-TNF biologics between the patients with and without sleep disturbances.

**Table 1:** Clinical characteristics of inflammatory bowel disease patients with or without sleep disturbances

	Sleep disturbances (−)	Sleep disturbances (+)	*p* value
	(N = 76)	(N = 60)	
Age (years)	43.8 ± 15.3	41.5 ± 20.6	0.48
Male sex (%)	54.3	56.7	0.61
Body mass index (kg/m^2^)	21.6 ± 2.7	21.2 ± 3.6	0.57
Alcohol drinking (%)	26.3	31.7	0.57
Smoking (%)	6.6	16.7	0.10
Caffeine intake (%)	53.9	41.7	0.17
IBD type			
UC	68.4	60.0	0.37
CD	31.6	40.0	
Disease duration (years)	11.7 ± 8.5	10.9 ± 8.8	0.57
Active disease (%)	14.5	18.3	0.64
HBI	1.08 ± 2.33	1.68 ± 2.90	0.42
pMayo score	1.19 ± 1.66	1.74 ± 1.96	0.18
Prior IBD surgery (%)	47.4	53.3	0.61
Medication used			
Steroids (%)	2.6	5.0	0.65
5-aminosalicylates (%)	78.9	78.3	1.00
Immunomodulators (%)	28.9	13.3	0.04
Anti-TNF biologics (%)	27.6	23.3	0.69
Sleep medications (%)	2.6	25.0	<0.01

Data are expressed as mean ± SD, or frequency. *IBD* inflammatory bowel disease, *UC* ulcerative colitis, *CD* Crohn’s disease, *HBI* Harvey-Bradshaw Index, *pMayo* partial Mayo score, *TNF* tumor necrosis factor

### Association between sleep disturbances and HR-QOL among IBD patients

**Fig. 3:**
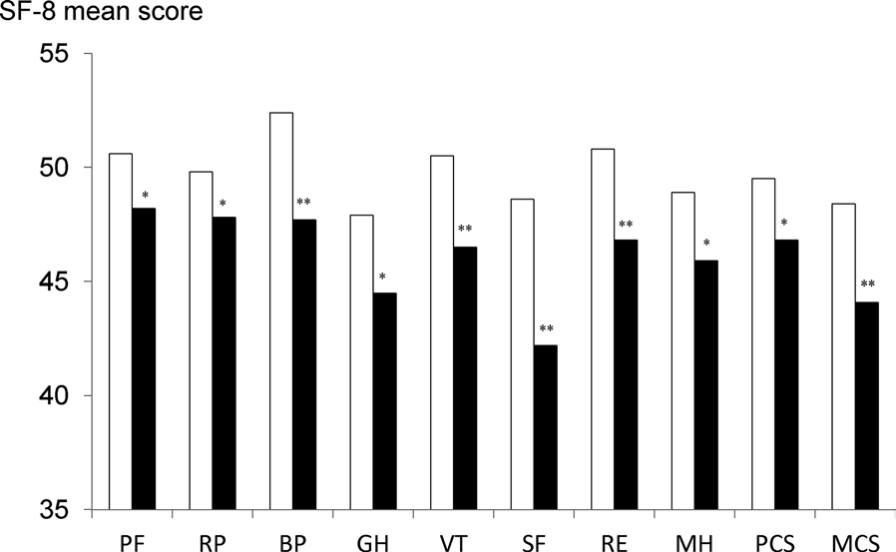
Health-related quality of life score according to the presence or absence of sleep disturbances in IBD patients. All scores, as well as two summary component scores, were significantly lower in patients with sleep disturbances than patients without sleep disturbances. *Data* are represented as mean. *White bars* and *black bars* are used to represent the mean scores of patients without sleep disturbances and patients with sleep disturbances, respectively. *p < 0.01, **p < 0.05 versus patients without sleep disturbances. PF physical functioning; RE, role physical; BP bodily pain; GH general health perception; VT vitality; SF, social functioning; RE role emotional; MH mental health; PCS physical component summary; MCS mental component summary

SF-8 scores for IBD patients with and without sleep disturbances are shown in Fig. 3. All scores from HR-QOL domains as well as summary scores (PCS and MCS) were significantly lower in patients with sleep disturbances in comparison to scores form the group of patients without sleep disturbances, suggesting that IBD patients with sleep disturbances experienced poorer HR-QOL compared to those without sleep disturbances.

### Risk factors for disease flare within 1 year

Eighty-five patients (62.5 %) displayed stable disease condition within 1 year, while 51 patients (37.5 %) had disease flare: 35 (39.8 %) out of 88 patients with UC and 16 (33.3 %) out of 48 with CD. Clinical characteristics of IBD patients with and without disease flare within 1 year from enrollment in the study are shown in Table 2. Among them, only the prevalence of sleep disturbances in the patients with disease flare was significantly higher (60.8 %) than that in the patients without disease flare (34.1 %). There were no significant differences in age, sex, BMI, alcohol or caffeine intake, smoking, IBD type, disease duration, activity, and medications between the two groups. Univariate and multivariate analyses performed to identify the risk factors for disease flare within 1 year are shown in Table 3. Only the sleep disturbances factor was significantly associated with disease flare (OR 2.69, 95 % CI 1.14–6.34, P = 0.02). Other factors including age, disease activity and duration, and current use of medications were not associated with disease flare. The multivariate analysis indicated the presence of sleep disturbances as a significant risk factor for disease flare within 1 year (OR 3.09, 95 % CI 1.47–6.43) after adjusting for age, disease duration, and presence of sleep disturbances.

**Table 2:** Clinical characteristics of the patients with or without disease flare within one year from enrollment

	Disease flare (−)	Disease flare (+)	*p* value
	(N = 85)	(N = 51)	
Age (years)	43.3 ± 17.2	41.8 ± 18.9	0.62
Male sex (%)	52.9	54.9	0.86
Body mass index (kg/m^2^)	21.5 ± 3.0	21.4 ± 3.3	0.85
Alcohol drinking (%)	25.9	31.7	0.43
Smoking (%)	9.4	13.7	0.57
Caffeine intake (%)	47.1	51.0	0.72
IBD type			
UC	62.4	68.6	0.58
CD	37.6	31.4	
Disease duration (years)	10.7 ± 7.5	12.5 ± 10.2	0.29
Active disease (%)	11.8	23.5	0.09
Prior IBD surgery (%)	48.2	52.9	0.72
Medication used			
Steroids (%)	3.5	3.9	1.00
5-aminosalicylates (%)	75.3	84.3	0.28
Immunomodulators (%)	23.5	19.6	0.67
Anti-TNF biologics (%)	29.4	19.6	0.23
Sleep medications (%)	8.2	19.6	0.06
Sleep disturbances (%)	34.1	60.8	<0.01

Data are expressed as mean ± SD, or frequency. *IBD* inflammatory bowel disease, *UC* ulcerative colitis, *CD* Crohn’s disease, *TNF* tumor necrosis factor

**Table 3:** Risk factors for disease flare within one year

Factors	Univariate			Multivariate^a^		
	OR	95 % CI	*p* value	OR	95 % CI	*p* value
Age (per 1 year)	0.97	0.95–1.00	0.09	0.99	0.96–1.01	0.27
Male sex	0.70	0.30–1.81	0.51			
Body mass index (per 1 kg/m^2^)	1.01	0.89–1.14	0.87			
Alcohol drinker	1.19	0.49–2.92	0.70			
Smoker	1.93	0.49–7.62	0.35			
Caffeine intake	1.02	0.45–2.30	0.97			
IBD type, CD	0.65	0.21–2.00	0.46			
Disease duration (per 1 year)	1.05	0.99–1.11	0.11	1.04	0.99–1.10	0.09
Active disease	1.87	0.65–5.40	0.25			
Prior IBD surgery	1.21	0.60–2.42	0.60			
Current medication use						
Steroids	0.96	0.12–7.80	0.97			
5-aminosalicylates	1.82	0.56–5.86	0.31			
Immunomodulators	1.37	0.52–3.65	0.52			
Anti-TNF biologics	0.62	0.18–2.10	0.44			
Sleep medications	1.75	0.46–6.69	0.42			
Sleep disturbances	2.69	1.14–6.34	0.02	3.09	1.47–6.43	<0.01

Data are expressed as mean ± SD, or frequency. *IBD* inflammatory bowel disease, *UC* ulcerative colitis, *CD* Crohn’s disease, *TNF* tumor necrosis factor, *OR* odds ratio, *CI* confidence intervals

^a^ Adjusted for age, disease duration, and sleep disturbances

## Discussion

Our results show that approximately half of all the IBD patients experienced sleep disturbances as assessed by the PSQI, and their HR-QOL was more impaired than that of IBD patients without sleep disturbances. Use of sleep medication was higher, while use of immuno modulators was lower, in patients with sleep disturbances compared to those without sleep disturbances. There were no differences in other clinical characteristics such as IBD type, disease duration, and disease activity between IBD patients with and without sleep disturbances. We found disease flare in 37.5 % of IBD patients within 1 year from enrollment, and we identified the presence of sleep disturbances as a significant risk factor for disease flare among the Japanese IBD patients.

An internet survey performed on the Japanese population showed that IBD patients, especially those with CD, had a poorer QOL, as assessed by SF-8, than healthy controls (Matsumoto et al. 2015). Although our study did not include healthy controls, all eight items of the SF-8 and two summary component scores were significantly lower in IBD patients with sleep disturbances than those without sleep disturbances, suggesting that sleep disturbances caused poorer HR-QOL among IBD patients. Similarly, Ranjbaran et al. reported that poorer sleep quality assessed by PSQI was positively correlated with poorer QOL assessed by the IBD Quality of Life Scale (Ranjbaran et al. 2007a). Taken together, our results indicate that the presence of sleep disturbances plays a significant role in the poor HR-QOL outcome observed in IBD patients.

The mechanisms underlying the association between sleep disturbances and IBD should be discussed. Although IBD-related nocturnal symptoms alter normal sleep conditions (Gingold-Belfer et al. 2014), our results showed no differences in HBI and pMayo scores between patients with and without sleep disturbances, suggesting that disease activity was not associated with sleep disturbances. This is further supported by another study that showed that IBD patients with inactive disease also had significant sleep disturbances (Ranjbaran et al. 2007a). However, several studies revealed a strong association between disease activity and sleep disturbances in IBD (Graff et al. 2011; Ali et al. 2013; Ananthakrishnan et al. 2014). Graff et al. reported that 77 % of IBD patients with active disease and 49 % of those with inactive disease experienced poor sleep as measured with the PSQI in a population-based study of 318 IBD patients (Graff et al. 2011). Further studies to investigate whether disease activity of IBD affects sleep in Japanese IBD patients are required. Use of medications such as corticosteroids is known to impair sleep; however, we found no difference in steroid use among the two patients groups examined in the present study. The reason why the use of immuno modulators was more elevated in patients without sleep disturbances than in those with sleep disturbances is presently unknown.

Our study identified sleep disturbances as a risk factor for IBD flare-ups. A recent study showed that sleep disturbances were associated with an increased risk of disease flares in CD, but not in UC (Ananthakrishnan et al. 2013). The group of CD patients with impaired sleep showed a twofold increase in risk of active disease during 6 months. In the present study, there were no differences in the risk of disease flares between CD and UC. The lack of consistency between our results and Ananthakrishna’s results may be attributed to the small sample size pf the present study and the racial/ethnic differences between the subjects analyzed in the two studies.

Sleep is associated with immune system activity; cytokines such as tumor necrosis factor-α (TNFα) and interleukin (IL)-6 directly induce sleep (Ranjbaran et al. 2007b) while sleep deprivation increases circulating inflammatory cytokine levels (Swanson et al. 2011; Parekh et al. 2015). Since these cytokines play an important role in the pathogenesis of IBD (Zisapel 2007; Bryant et al. 2004), the association between IBD and sleep disturbances may be bidirectional due to the elevated inflammatory cytokine levels induced by sleep alterations. Tang et al. reported that both acute and chronic sleep deprivation exacerbates dextran sodium sulfate-induced colitis in a mouse model of colitis (Tang et al. 2009). The observation that sleep deprivation promotes a worsening of colitis might explain why the presence of sleep disturbances was found to be a significant risk factor for IBD disease flare in our study.

We must acknowledge some limitations of the present study. First, the sample size was relatively small for a single referral hospital. Second, IBD patients with sleep disturbances were using more sleep medications than IBD patients without sleep disturbances. However, the univariate analysis revealed that the use of sleep medications was not associated with disease flare within 1 year from enrollment. Third, a consensus on the definition of disease flare of IBD has not been reached yet. The definition of disease flare, as well as the relatively short follow-up period, we adopted in the present study might have affected the present results. A long-term follow up study is deemed necessary to further confirm our results. Fourth, several studies showed that both a depressive mode and anxiety disorders are associated with sleep disturbances (Benhayon et al. 2013) and IBD flare-ups (Pariante and Miller 2001). Future studies should include these factors.

## Conclusions

In conclusion, sleep disturbances are common in Japanese IBD patients and they are associated with poor HR-QOL. Since the presence of sleep disturbances was found to be a significant risk factor for IBD flare-ups within 1 year, we advise physicians to pay more attention to sleep disturbances in IBD patients.

## Abbreviations

IBD: inflammatory bowel disease
CD: Crohn’s disease
UC: ulcerative colitis
HR-QOL: health-related quality of life
PSQI: Pittsburg sleep quality index
SF-8: the 8-item short-form health survey
PF: physical functioning
RE: role physical
BP: bodily pain
GH: general health perception
VT: vitality
SF: social functioning
RE: role emotional
MH: mental health
PCS: physical component summary
MCS: mental component summary
HBI: Harvey-Bradshaw index
pMayo: partial Mayo score
